# Glycans housed by a bacteriophage enable rapid identification of glycan recognition patterns

**DOI:** 10.1038/s42003-021-02474-7

**Published:** 2021-08-12

**Authors:** Theam Soon Lim, Karli Montague-Cardoso

**Affiliations:** 1 Institute for Research in Molecular Medicine, Universiti Sains Malaysia, Pulau Pinang, Malaysia; 2 Communications Biology, https://www.nature.com/commsbio

## Abstract

Glycans are a major composition of the cell surface that interacts with the surrounding environment. The ability to carry out glycan-binding profile studies has been mainly done with glycan arrays. However, glycan arrays are not easily adaptable for cell surface and in vivo glycan recognition assays. The Liquid Glycan Array (LiGA) reported recently by Sojitra et al. is an alternative glycan recognition assay that employs DNA barcoding, bioorthogonal ligation and deep sequencing technology. In LiGA, barcoded M13 virions are used to present glycans to allow rapid identification of binding partners based on sequence identity. This physical link between the glycan to the DNA sequence fitted in the phage genome provides an ingenious approach to maneuver glycan binding profile studies in various conditions.

The surface of cells has a rich layer of glycans that are a combination of glycoproteins and glycolipids. These glycans constitute a major molecular interface between cells and their environment. The glycome of each cell is made up of varying glycan structures which functions as a cellular signature. This unique pattern is recognized by complementary glycan-binding proteins (GBPs) which can translate these patterns to function. This is evident in the immune system where it is used for pathogen recognition and regulates inflammatory responses. A major challenge when interrogating these glycan–GBP interaction patterns is the number of binding sites and spatial presentation of glycans. Although conventional approaches like array technology allows multivalent presentation of the glycans, it does suffer from signal resolution when using a single glycan and is limited when studying cross talk of glycan and GBP. In addition, the array system is incompatible for in vivo studies, which is a major drawback for cell-surface GBP studies.

In a recent study, Sojitra et al. describe a new approach, which they have coined as Liquid Gycan Array (LiGA). This approach leverages on the technologies of DNA barcoding, deep-sequencing and biorthogonal conjugation of glycans to generate glycan presenting phage virions^1^. The authors used virions of a filamentous bacteriophage as the solid phase with the 2700 copies of the pVIII coat protein acting as the anchor to attach the glycans. As the amount of the pVIII protein on the surface of a virion is high, this allows multiple copies of the glycans to be attached to each phage particle. The attachment is done by using dibenzocyclooctyne to functionalize the protein on the surface of the virioin to attach the azido-modified glycans. As specific DNA barcodes are introduced at the pIII gene, each virion population would have a specific DNA barcode. Each population of different DNA barcoded virions will each be conjugated with a specific glycan. This allows for a physical link between the specific glycan to each virion harboring a specific DNA code. Therefore, the binding interaction of each glycan harboring virion with a specific GBP can be rapidly identified by deep-sequencing analysis. The approach was shown to successfully identify critical glycan-binding information about glycans and cell-displayed GBP either in vitro or in vivo.

This approach shows a significant improvement to the existing glycan array and glycophage methods for glycan–GBP interaction studies. With the establishment of a catalogue of possible glycans on phage virions, the analysis and identification of interacting glycans can be done rapidly in vitro, on cell surfaces and in vivo for various diseases. This will help pave the way to better understand the biological functions of glycans.
